# Bronchial epithelial cells are rendered insensitive to glucocorticoid transactivation by transforming growth factor-β1

**DOI:** 10.1186/1465-9921-15-55

**Published:** 2014-05-01

**Authors:** Christine R Keenan, Josephine SL Mok, Trudi Harris, Yuxiu Xia, Saad Salem, Alastair G Stewart

**Affiliations:** 1 Lung Health Research Centre, Department of Pharmacology and Therapeutics, University of Melbourne, Grattan St., Parkville, VIC Australia; 2 Department of Pharmacology, University of Malaya, Kuala Lumpur, Malaysia

**Keywords:** Steroid resistance, Inflammation, GRE, Smad4, Epigenetics

## Abstract

**Background:**

We have previously shown that transforming growth factor-beta (TGF-beta) impairs glucocorticoid (GC) function in pulmonary epithelial cell-lines. However, the signalling cascade leading to this impairment is unknown. In the present study, we provide the first evidence that TGF-beta impairs GC action in differentiated primary air-liquid interface (ALI) human bronchial epithelial cells (HBECs). Using the BEAS-2B bronchial epithelial cell line, we also present a systematic examination of the known pathways activated by TGF-beta, in order to ascertain the molecular mechanism through which TGF-beta impairs epithelial GC action.

**Methods:**

GC transactivation was measured using a Glucocorticoid Response Element (GRE)–Secreted embryonic alkaline phosphatase (SEAP) reporter and measuring GC-inducible gene expression by qRT-PCR. GC transrepression was measured by examining GC regulation of pro-inflammatory mediators. TGF-beta signalling pathways were investigated using siRNA and small molecule kinase inhibitors. GRα level, phosphorylation and sub-cellular localisation were determined by western blotting, immunocytochemistry and localisation of GRα–Yellow Fluorescent Protein (YFP). Data are presented as the mean ± SEM for *n* independent experiments in cell lines, or for experiments on primary HBEC cells from *n* individual donors. All data were statistically analysed using GraphPad Prism 5.0 (Graphpad, San Diego, CA). In most cases, two-way analyses of variance (ANOVA) with Bonferroni post-hoc tests were used to analyse the data. In all cases, P <0.05 was considered to be statistically significant.

**Results:**

TGF-beta impaired Glucocorticoid Response Element (GRE) activation and the GC induction of several anti-inflammatory genes, but did not broadly impair the regulation of pro-inflammatory gene expression in A549 and BEAS-2B cell lines. TGF-beta-impairment of GC transactivation was also observed in differentiated primary HBECs. The TGF-beta receptor (ALK5) inhibitor SB431541 fully prevented the GC transactivation impairment in the BEAS-2B cell line. However, neither inhibitors of the known downstream non-canonical signalling pathways, nor knocking down Smad4 by siRNA prevented the TGF-beta impairment of GC activity.

**Conclusions:**

Our results indicate that TGF-beta profoundly impairs GC transactivation in bronchial epithelial cells through activating ALK5, but not through known non-canonical pathways, nor through Smad4-dependent signalling, suggesting that TGF-beta may impair GC action through a novel non-canonical signalling mechanism.

## Introduction

Glucocorticoids are the most effective class of anti-inflammatory drugs available. However, several chronic inflammatory diseases appear to be inherently resistant to glucocorticoid therapy [1]. Even in disease states where glucocorticoids are usually effective, patient sensitivity to glucocorticoid action varies dramatically. Indeed, different tissues from the same patient may even differ in sensitivity, suggesting that it is the chronic inflammatory microenvironment that is responsible for localized resistance to glucocorticoid action [2,3]. Several biochemical alterations have been associated with glucocorticoid resistance including increased levels of cytokines (TNFα, IFNγ, IL-2, IL-4, IL-13, IL-17, IL-27, MIF), oxidative and nitrative stress, and the presence of denatured collagen [1,4,5]. Despite the identification of these associations, the elucidation of the activation of downstream signalling pathways and the resultant impairment of the action of the glucocorticoid receptor, glucocorticoid resistance remains a major obstacle in the treatment of chronic inflammatory diseases.

Appreciation for the role of airway epithelial cells as a target for glucocorticoid therapy is ever increasing. The epithelium is the site of deposition for inhaled glucocorticoids, and as such can be exposed to much higher concentrations of glucocorticoid than any other cell type in the airway. A corollary of the importance of airway epithelial cells as a target for glucocorticoid activity is that it is also a key cell type in which glucocorticoid resistance may develop. There have been limited numbers of studies investigating the molecular mechanisms of resistance in epithelial cells, as opposed to the extensive attention paid to inflammatory cell types [4]. It is known, however, that inflammatory stimuli such as TNFα, and mitogens such as foetal calf serum (FCS), can induce resistance to glucocorticoid transactivation in epithelial cells [6]. Similarly, IL-17A has been found to induce resistance to glucocorticoid regulation of IL-8 production in epithelial cells [7]. Very recently, rhinovirus infection has also been shown to cause glucocorticoid resistance in airway epithelium through the activation of NFκB and JNK and impaired nuclear entry of GRα [8]. The epithelium is therefore gaining increasing attention as a cell type in which glucocorticoid resistance can develop.

We have previously shown that transforming growth factor-beta (TGF-β) impairs GRE-dependent transactivation and glucocorticoid regulation of interleukin-6 and interleukin-8 production in the A549 lung adenocarcinoma-derived epithelial cell line, in association with decreased nuclear translocation of GRα [9]. This resistance was not restricted to the A549 cell line, as TGF-β was also shown to impair glucocorticoid regulation of thrombin and IL-1 stimulated GM-CSF release from BEAS-2B bronchial epithelial cells. However, the precise molecular mechanism(s) through which TGF-β impairs glucocorticoid function is yet to be established in either cell line, or in primary bronchial epithelial cells.

TGF-β is renowned for its pleiotropic actions on a wide variety of signalling pathways. Depending on the cellular context, TGF-β can activate Smad proteins resulting in transcriptional regulation, and it can activate a wide variety of non-canonical pathways, such as MAP kinase pathways, phosphoinositide 3-kinase (PI3K) and Rho pathways. The NFκB pathway is also activated through increased IKK activity [10]. In the present study, we provide the first evidence that TGF-beta impairs GC action in the ‘gold standard’ in vitro epithelial model: differentiated primary air-liquid interface (ALI) human bronchial epithelial cells (HBECs). Furthermore, using the BEAS-2B bronchial epithelial cell line, we also present a systematic examination of the known pathways activated by TGF-beta, in order to ascertain the molecular mechanism through which TGF-beta impairs epithelial GC action.

## Materials and methods

### Cell culture

A549 lung adenocarcinoma and BEAS-2B bronchial epithelial cell lines were cultured as described [9]. Primary HBECs were purchased from Lonza (Waverley, Australia) and cultured using B-ALI™ BulletKit™ (Lonza) according to manufacturer’s instructions.

### Air-liquid interface differentiation of primary HBECs

Primary HBECs were differentiated on fibrillar collagen-coated 24-well Corning® Transwell® inserts (Sigma #CLS34700, MO, USA) by culture at air-liquid interface for 4 weeks. Cell differentiation was confirmed through visualisation of beating cilia and measurement of trans-epithelial electrical resistance (TEER) using an EVOM2 Voltohmmeter (WPI, Sarasota, FL). Cells were treated with 40 pM TGF-β or control for 24 h, then stimulated with 100 nM dexamethasone (Dex), 100 nM budesonide (Bud) or vehicle control for 4 h after which total RNA was extracted and GC-inducible gene expression was measured by qRT-PCR.

### Transfection of epithelial cells

BEAS-2B cells were co-transfected using Lipofectamine® 2000 (Invitrogen, Carlsbad, CA) as previously described [9]. Plasmid vectors: pGRE-SEAP (Clontech Laboratories Inc., Mountain View, CA), pSM22-Luc [11], GRα-YFP construct (a kind gift from Prof John Cidlowski [12]). pmax-GFP (Amaxa, Lonza, Waverley, Aus) and pGL3-Luc (Promega, Alexandria, NSW, Aus) were used as internal transfection controls. Transfected cells were treated with TGF-β (4–100 pM) for 24 h prior to the addition of dexamethasone (Dex, 0.1-100 nM) or vehicle for 2 h. GFP and YFP expression were imaged using live-cell fluorescence microscopy using a Leica DMI6000B microscope. Supernatants were collected for measurement of secreted human placental alkaline phosphatase (SEAP) using a chemiluminescence kit (Roche Applied Science, NSW, Aus). Luciferase expression was determined from lysates prepared by scraping cells in 25 mM tris-phosphate buffer (pH 7.8) containing 10% glycerol, 1% triton X-100, 1 mg/mL BSA, 2 mM EDTA, 2 mM DTT. Cell lysate luciferase luminescence activity was determined by reagent buffer consisting of 20 mM Tris (pH 7.8), 33.3 mM DTT, 8 mM MgCl_2_, 0.13 mM EDTA, 0.47 mM Beetle Luciferin (Promega #E1601). siRNA targeting exon 7 of Smad4 (sense: 5′-GCCAUAGUGAAGGACUGUUtt-3′, Shanghai Genepharma, Shanghai, China), was transfected using Lipofectamine RNAiMAX (Invitrogen) as described previously [13].

### RNA extraction and RT-qPCR

Total RNA was purified from A549 and BEAS-2B cell lines using TRIzol (Invitrogen, Doncaster, VIC, Australia), and from differentiated HBECs using RNeasy miniprep kit (Qiagen, Doncaster, VIC, Australia). RNA (100 ng) was reverse-transcribed in a 5 μL reaction using the High Capacity cDNA Reverse Transcription Kit (Applied Biosystems, Scoresby, VIC, Australia) then diluted with 145 μL DEPC-treated H_2_O. Real-time PCR was performed using ABI Prism 7900HT sequence detection system as previously described [9]. The generation of specific PCR products was confirmed by dissociation curve analysis. Primer sequences (Table 1) were obtained either from literature, or designed using Primer Express software (Applied Biosystems) with mRNA sequences from the National Centre for Biotechnology Information (http://www.ncbi.nlm.nih.gov).

**Table 1:** Primer Sequences for RT-PCR

**Gene product**	**Forward primer**	**Reverse primer**
18S	CGC CGC TAG AGG TGA AAT	TCT TGG CAA ATG CTT TCG CTC
CDKN1C	TCT GAT CTC CGA TTT CTT CG	CTC TTT GGG CTC TAA ATT GG
ENaCα	AGC ACA ACC GCA TGA AGA C	TGA GGT TGA TGT TGA GGC TG
FKBP5	AGA TTG AGC TCC TTG ATT TC	TGA ATA TCC CTC TCC TTT CC
GILZ	TCC TGT CTG AGC CCT GAA GAG	AGC CAC TTA CAC CGC AGA AC
GRβ	TCA ACT GAC AAA ACT CTT GG	AGT GCA CAT AAT CTT CTT TTT CTC A
IκBα	TAC CAA CTA CAA TGG CCA CAC G	TAG CCA TGG ATA GAG GCT AAG TGT AGA
MKP-1	CCA CAA GGC AGA CAT CAG CTC	TCT ATG AAG TCA ATG GCC TCG TT
PAI-1	TCA GGC TGA CTT CAC GAG TCT TT	CTG CGC GAC GTG GAG AG
SERPINA3	TCA AGA CAA GAT GGA GGA AG	TCA CCT ATC TCT CTG AAC TC
SLPI	GCA TCA AAT GCC TGG ATC CT	GCA TCA AAC ATT GGC CAT AAG TC
SMAD4	CCA GGA TCA GTA GGT GGA AT	GTC TAA AGG TTG TGG GTC TG
Tektin-1	ATT ACA GCT CTT GAA AAG GC	GGG CTA AAG TTT CCT TCA ATC

### Quantification of IL-6 and PGE_2_ levels in supernatants

Supernatant IL-6 was assayed by Enzyme-linked Immunosorbent Assay (ELISA) (Becton Dickinson, Heidelberg, Germany) [9]. The level of PGE_2_ was measured by radioimmunoassay (RIA) according to previous studies [14] using commercial anti-PGE_2_ antiserum (Sigma #P5164).

### GR expression, phosphorylation and sub-cellular localization

Glucocorticoid receptor expression and phosphorylation state was investigated by western blot analysis as described [13] using polyclonal antibodies directed against GRα, phospho-GR(Ser203), phospho-GR(Ser211), phospho-GR(Ser226) (Santa Cruz Biotechnology). The levels of protein and β-actin (β-actin mouse monoclonal: Abcam, Cambridge, UK) were used to control for loading. GR subcellular localization was determined by GRα-YFP transfection (described above) and immunocytochemistry against GRα as described [9].

### Cell viability

Cell viability was assessed using the Trypan blue exclusion method [13]. Cell viability was determined to be >95% at the conclusion of all experimental treatment periods in these studies.

### Statistical analyses

Data are presented as the mean ± SEM for *n* individual experiments. All data were statistically analysed using GraphPad Prism 5.0 (Graphpad, San Diego, CA). In most cases, two-way analyses of variance (ANOVA) with Bonferroni *post hoc* tests were used to analyse the data. A P value of <0.05 was considered to be statistically significant.

## Results

### TGF-β impairs glucocorticoid transactivation in BEAS-2B cells

**Figure 1:**
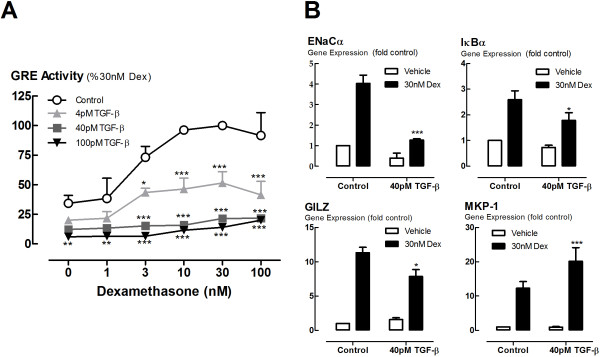
**Effect of TGF-**β **on glucocorticoid transactivation.** BEAS-2B cells were incubated with TGF-β (4-100pM) for 24 h before stimulation by dexamethasone (1-100 nM). **(A)** GRE activity was measured in BEAS-2B cells transiently transfected with a GRE-SEAP reporter construct, incubated with TGF-β (4-100 pM), then stimulated with dexamethasone for a further 24 hours. The level of SEAP in the supernatants was expressed as a percentage the level induced in response to 30nM dexamethasone. **(B)** Glucocorticoid-inducible gene expression in non-transfected cells. BEAS-2B cells were incubated with TGF-β (40 pM) for 24 h before stimulation by dexamethasone (30 nM) for 4 h after which RNA was extracted and analysed by qRT-PCR. Gene expression is expressed as fold change from control. Data are presented as mean and SEM for *n* = 4 independent experiments. *P < 0.05, **P < 0.01, *P < 0.001 *c.f.* Control **(A)**, 30 nM Dex **(B)**.

In BEAS-2B cells transfected with a plasmid bearing a GRE-controlled SEAP expression vector, incubation with TGF-β potently and extensively inhibited Dex-induced GRE activity with 4 pM sufficient to inhibit the maximum response by 50%, and complete inhibition observed at 40 pM TGF-β (Figure 1A). The GRE within the GRE-SEAP construct may respond differently to the GREs within the sequences of endogenous GRE-regulated genes in their orthotopic genomic context. Thus, measurement of the mRNA expression of a variety of GRE-inducible genes was used to assess the effect of TGF-β on dexamethasone-stimulated transactivation in the BEAS-2B cell line. Of the panel of genes assessed, the expression of most were markedly impaired. For example, the genes encoding epithelial sodium channel-α subunit (ENaCα), NFκB inhibitor-α (IκBα), glucocorticoid-inducible leucine zipper (GILZ) (Figure 1B), annexin 1 (ANXA1) and secretory leukocyte protease inhibitor (SLPI) (data not shown) were all impaired. The expression of some genes, however, was unchanged or enhanced at the time-point measured. For example, the expression of the gene encoding MAP kinase phosphatase 1 (MKP-1) was enhanced by TGF-β conditioning prior to dex exposure (Figure 1B).

### TGF-β does not cause widespread impairment of glucocorticoid regulation of cytokine production in epithelial cell lines

**Figure 2:**
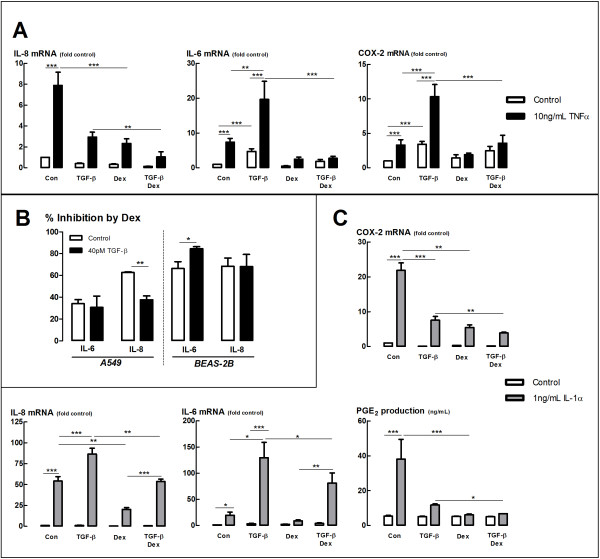
**Effect of TGF-**β **on glucocorticoid transrepression. (A)** BEAS-2B cells were incubated with TGF-β (40pM) for 24 h, then incubated with dexamethasone (30 nM) for 30 min prior to stimulation with TNFα (10 ng/mL) for 4 h. RNA was then extracted and expression was analysed by qRT-PCR and expressed as fold change from control. **(B)** Data from **(A)** and **(C**) expressed as percentage inhibition by dexamethasone in the presence and absence of TGF-β. **(C)** A549 cells were incubated with TGF-β (40 pM) for 24 h, then incubated with dexamethasone (10 nM) for 30 min prior to stimulation with IL-1α (1 ng/mL) for 4 h (Gene expression) or 24 h (PGE_2_ determination). Gene expression was determined as in **(A)**, PGE_2_ levels were determined by radioimmunoassay. Data are presented as mean and SEM for *n* = 3-4 independent experiments. *P < 0.05, **P < 0.01, ***P < 0.001.

In order to measure the effect of TGF-β on GC transrepression, we examined the glucocorticoid regulation of pro-inflammatory gene expression. In the BEAS-2B cell line, we examined the expression of genes widely accepted to be regulated by transrepression. We found, as expected, that the pro-inflammatory cytokine TNFα significantly induced the expression of the genes encoding IL-6 and IL-8 in a dexamethasone-sensitive manner (Figure 2A). TGF-β alone induced the expression of IL-6 mRNA and further enhanced the induction by TNFα. Nevertheless, this induction of IL-6 mRNA was suppressed by dexamethasone, and the presence of TGF-β did not significantly reduce the level of inhibition by dex (Figure 2A,B). A similar pattern of results was observed for the regulation of COX-2 mRNA (Figure 2A). Although TGF-β inhibited the expression of IL-8 mRNA (Figure 2A), dexamethasone was similarly effective in inhibiting IL-8 expression in the presence and absence of TGF-β (Figure 2B).

We had previously shown that dexamethasone regulation of IL-8 production is impaired by TGF-β in the A549 cell line [9]. However, as this was not observed in the BEAS-2B cell line, we decided to further examine the effect of TGF-β on glucocorticoid repression of cytokine expression in the A549 cell line to establish whether the TGF-β effect is specific to IL-8 regulation, or extends to the breadth of transrepressional impacts in this cell line. IL-1α significantly induced IL-6 and IL-8 mRNA in a dexamethasone-sensitive manner in the A549 cell line (Figure 2C). TGF-β enhanced the IL-1α stimulation of both IL-6 and IL-8 mRNA (Figure 2C). Dexamethasone significantly suppressed the enhanced IL-6 and IL-8 mRNA in the presence of TGF-β (Figure 2C). However, the extent of the suppression of IL-8 mRNA was reduced in the presence of TGF-β (Figure 2B). Interestingly, dexamethasone was similarly effective at inhibiting IL-6 mRNA (and IL-6 protein release, data not shown) in the presence and absence of TGF-β in the A549 cell line, similar to the BEAS-2B cell line (Figure 2B). Dexamethasone regulation of PGE_2_ production and COX-2 mRNA expression was also similar in the presence and absence of TGF-β in the A549 cell line, despite an inhibition of PGE_2_ production and a stimulation of COX-2 expression by TGF-β alone (Figure 2C).

### TGF-β impairs glucocorticoid transactivation in differentiated primary HBECs

**Figure 3:**
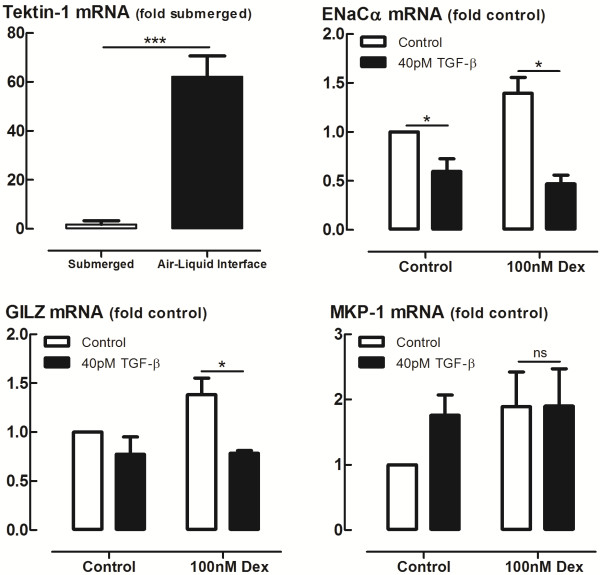
**Effect of TGF-βon glucocorticoid transactivation in air-liquid-interface (ALI) differentiated primary human bronchial epithelial cells.** Primary human bronchial epithelial cells (HBECs) were differentiated at air-liquid interface for 28 days, treated with 40 pM TGF-β or control for 24 h, then stimulated with 100 nM dexamethasone (Dex) or vehicle control for 4 h after which total RNA was extracted and GC-inducible gene expression was measured by qRT-PCR. Gene expression is expressed as fold change from control. Data are presented as mean and SEM from differentiated cells from *n* = 4 individual donors. *ns* P > 0.05, *P < 0.05, **P < 0.01, *P < 0.001.

Primary HBECs met the criteria for ALI differentiation, including TEER values greater than 700 Ω/cm^2^ (data not shown) and increased Tektin-1 mRNA expression (Figure 3), a marker of ciliated cell differentiation. TGF-β impaired Dex-induced expression of several anti-inflammatory genes, including the genes encoding GILZ and ENaCα in ALI differentiated cells (Figure 3). Consistent with observations in epithelial cell lines, the expression of MKP-1 was not impaired following incubation with TGF-β (Figure 3). TGF-β also impaired budesonide-induced gene expression in a similar pattern (data not shown).

### TGF-β-induced impairment of glucocorticoid action is due to activation of activin-like kinase 5, but not due to activation of known non-canonical signalling pathways

**Figure 4:**
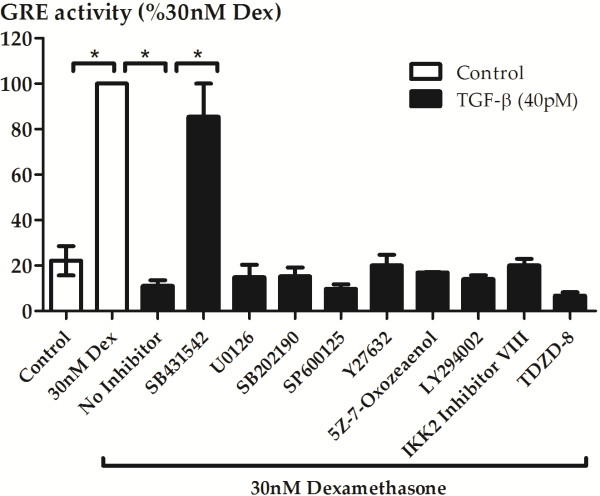
**Inhibition of known TGF-βnon-canonical signalling pathways does not prevent the impairment of GRE activity.** BEAS-2B cells transiently transfected with GRE-SEAP reporter construct were pre-incubated for 30 min with inhibitors (1 μM SB431542: TGF-β type 1 activin receptor-like kinase (ALK) receptors, 10 μM LY294002: non-selective PI3-kinase inhibitor, 1 μM U0126: MEK1/2 inhibitor, 1 μM SB202190: p38^MAPK^ inhibitor, 10 μM 5Z-7-oxozeaenol: TAK1 inhibitor, 1 μM SP600125: JNK inhibitor, 10 μM IKK2 inhibitor VIII: selective IKK2 inhibitor, 10 μM TDZD-8: selective GSK-3 inhibitor) prior to the addition of 40pM TGF-β for 24 h and stimulation with 30nM dexamethasone for a further 24 h. Supernatants were then collected for determination of SEAP level and results were then expressed as a percentage of the level induced in response to 30nM dexamethasone. Data are presented as mean and SEM for *n* = 3-4 independent experiments. *P < 0.05, **P < 0.01, ***P < 0.001 significantly different from control expression.

Impairment of glucocorticoid action in the A549 cell line was found to be associated with activation of the activin-like kinase 5 (ALK5/TGFβRI kinase) receptor, but the downstream signalling pathways activated have yet to be elucidated. We therefore investigated the involvement of the ALK5 receptor in the BEAS-2B cell line using the selective ALK5 inhibitor SB431542 (1 μM), and other commonly used and well-characterized small molecule inhibitors (1–10 μM based on our previous use [9,15] or the use of others [16-19]) of the known non-canonical signalling pathways of TGF-β to examine which one or more of the downstream pathways may be involved. Inhibition of ALK5 using SB431542 completely prevented TGF-β-induced impairment of GRE activation in the BEAS-2B cell line. However, inhibitors of the downstream non-canonical signalling pathways had no effect on TGF-β-induced GRE impairment (Figure 4). Since it is conceivable that activation of more than one downstream pathway is responsible for impairment of glucocorticoid action, multiple inhibitors were used to simultaneously block multiple pathways, but no effect was observed on TGF-β-induced GRE impairment (data not shown).

### TGF-β-induced impairment of glucocorticoid action is not dependent on Smad4

**Figure 5:**
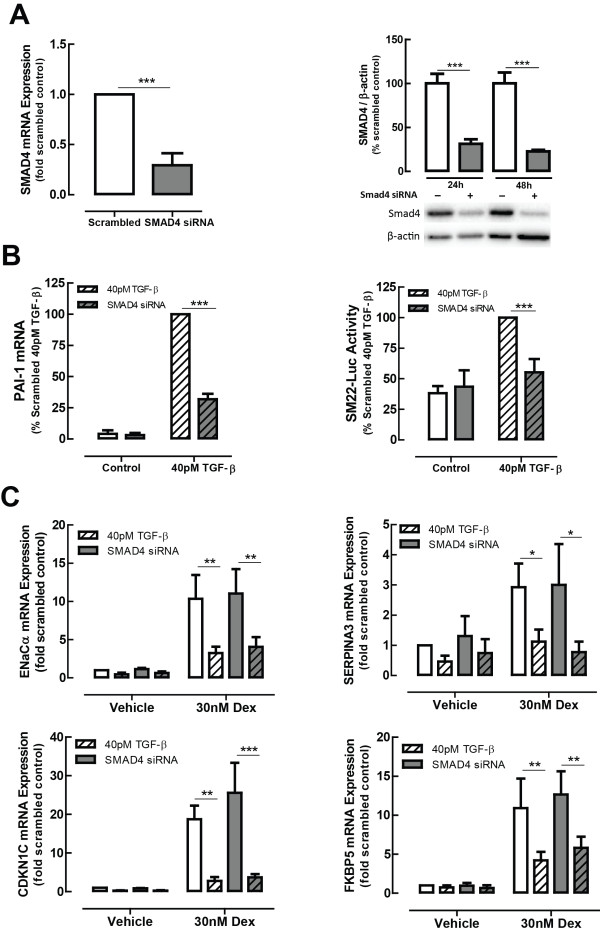
**Smad4 does not mediate TGF-**β **induction of glucocorticoid insensitivity.** Smad4 targeted siRNA reduces Smad4 expression **(A)**, Smad-dependent gene expression and the activity of the Smad-dependent promoter of SM22 **(B)**, but does not alter the level of impairment of GC-inducible gene activity by TGF-β **(C)**. BEAS-2B cells were transfected with scrambled control or Smad4-targeted siRNA, then incubated with TGF-β (40 pM) for 24 h, followed by stimulation with Dex (30 nM) for 4 h. Data are presented as mean and SEM for *n* = 4 independent experiments. *P < 0.05, **P < 0.01, ***P < 0.001.

Smad4-targeted siRNA was used to examine canonical TGF-β signalling, since this protein forms a unique, common point within canonical TGF-β signalling pathways. Smad4-targeted siRNA resulted in a knockdown of more than 60% which persisted throughout the experimental period (Figure 5A). A concomitant impairment of Smad-dependent gene expression was confirmed by measurement of PAI-1 expression, along with a complete impairment of TGF-β-induced SM22 promoter activity (Figure 5B). However, neither GC-induced gene expression, nor its impairment by TGF-β, was affected by Smad4 knockdown (Figure 5C).

### TGF-β-induced impairment is not associated with either impaired GRα expression, nuclear localization or altered GRα phosphorylation status in the BEAS-2B cell line

**Figure 6:**
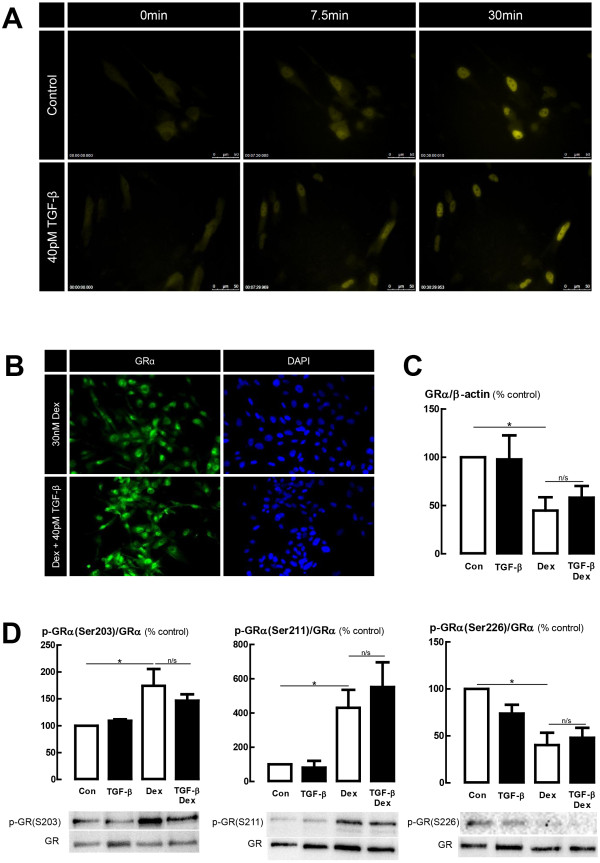
**TGF-βdoes not influence glucocorticoid-induced GRα nuclear localization. (A)** BEAS-2B cells transiently transfected with a GRα-YFP reporter construct were treated with TGF-β (40 pM) for 24 h then imaged before and after treatment with 30 nM dexamethasone (Dex), using time-lapse live-cell fluorescence microscopy (Leica DMI 6000B). Images were obtained every 30 sec for 30 min prior to Dex treatment, and for 2 h post-treatment. **(B)** Immunofluorescence of GRα in non-transfected BEAS-2B cells. Cells were incubated using an identical protocol to that in **(A)** except that at the end of the 2 h dexamethasone incubation, cells were fixed with 10% NBF then GRα immunolocalisation was probed with a FITC-labelled secondary antibody, and nuclei were co-stained with DAPI. Images presented are representative of four independent experiments. **(C,D)** Protein expression and phosphorylation state of GRα. Cell lysates from BEAS-2B cells were collected after 24 h treatment TGF-β (40pM), then 1 h treatment with 30nM dexamethasone. SDS-PAGE and immunoblotting using phospho-specific antibodies was performed, then the membrane was stripped and re-probed for total GRα expression for normalization. GRα expression was then normalized to β-actin expression. Data are presented as mean and SEM of 4 experiments. *P < 0.05, significantly different from control expression.

In the A549 cell line, TGF-β-induced glucocorticoid impairment was partially attributed to impaired nuclear localization of GRα. We therefore investigated the potential relevance of this mechanism in BEAS-2B cells using live cell fluorescence microscopy of cells transiently transfected with a GRα-YFP construct. Localization of GRα-YFP fluorescence indicated that TGF-β did not affect the rate or extent of GRα nuclear localization following dexamethasone treatment (Figure 6A, Additional file 1). This observation was confirmed by immunofluorescence staining of non-transfected cells where equivalent GRα immunoreactivity was observed in both nuclear and cytoplasmic compartments of TGF-β treated and control cells (Figure 6B).

Determination of GRα protein expression showed a significant reduction by 30 nM dexamethasone treatment, according with expectations based on previous studies in A549, BEAS-2B and HeLa epithelial cell lines [20,21]. However, treatment with TGF-β did not affect either the level, or the down-regulation in the presence of dexamethasone (Figure 6C). Similarly, dexamethasone treatment altered the phosphorylation state of GRα according to expectations [22] with an increase in phosphorylation at serine 203 and serine 211 observed, and a decrease in phosphorylation at serine 226. Treatment with TGF-β had no effect on either basal phosphorylation state or the dexamethasone-induced changes in phosphorylation. Up-regulation of the non-ligand binding splice variant of the glucocorticoid receptor, GRβ, has been proposed to impair glucocorticoid activity through inhibiting the actions of GRα [23-25] or through the recruitment of histone deacetylases (HDACs) [26]. qRT-PCR using validated primers to amplify GRβ did not produce a detectable product after 40 cycles of PCR for either control, dexamethasone-treated, or TGF-β treated cells.

### TGF-β-induced impairment of glucocorticoid action is not a result of epigenetic repression of gene transcription

**Figure 7:**
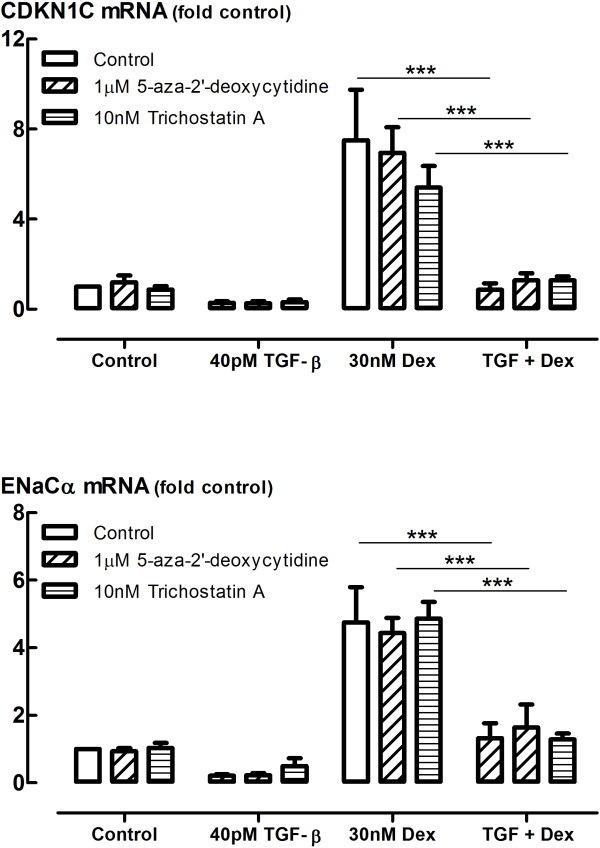
**Effect of inhibitors of DNMTs and HDACs on TGF-βimpairment of glucocorticoid-inducible gene expression.** Effect of DNA methyltransferase (DNMT) inhibitor 5-aza-2′-deoxycytidine and HDAC inhibitor trichostatin A on TGF-β impairment of glucocorticoid-inducible gene expression. BEAS-2B cells were incubated with inhibitors for 30 min prior to treatment with TGF-β (40 pM) for 24 h before stimulation by dexamethasone (30 nM) for 4 h after which RNA was extracted and analysed by qRT-PCR. Gene expression is expressed as fold change from control. Data are presented as mean and SEM for *n* = 3-4 independent experiments *P < 0.001.

Epigenetic modifications such as DNA methylation through DNA methyltransferase (DNMT) and histone deacetylation through histone deacetylase (HDAC) are known to cause repression of gene transcription. We therefore examined whether 5-aza-2′deoxycytidine, a DNMT inhibitor, or Trichostatin A, an HDAC inhibitor, can prevent the impairment of glucocorticoid transactivation by TGF-β. However, inhibitors of DNMT or HDAC had no effect on the dexamethasone-induced expression of the genes encoding cyclin-dependent kinase inhibitor 1C (CDKN1C) and ENaCα (SCNN1A), nor the impairment of gene transcription by TGF-β (Figure 7).

## Discussion

In this study, we have sought to ascertain the precise molecular mechanisms through which TGF-β impairs glucocorticoid action in epithelial cells. In order to successfully do this, it is necessary to first have a thorough understanding of the scope of impairment of glucocorticoid function. This study demonstrated that whilst TGF-β has a profound inhibitory effect on glucocorticoid transactivation, transrepressional mechanisms appear to remain intact. Since it is commonly accepted that glucocorticoids produce the majority of their anti-inflammatory effects through the repression of pro-inflammatory pathways [27], one might question whether the impairment of GC transactivation by TGF-β is a therapeutically relevant problem. One might even contend that TGF-β impairment of GC transactivation might be beneficial to the treatment of patients, since the multitude of side-effects from GC treatment [28] have been commonly thought to occur through transactivation mechanisms [29]. These dogmas, however, have never been universally accepted, and it is increasingly accepted that transactivational induction of anti-inflammatory genes such as Annexin 1, GILZ and MKP-1 are key to the anti-inflammatory actions of glucocorticoids [30,31]. Moreover, it has recently been reported that side effects of glucocorticoids are closely associated with transrepression of nGRE-containing genes, rather than transactivation of pGRE-containing genes [32]. We contend, therefore, that transactivation is an important anti-inflammatory mechanism of glucocorticoids, and the very potent effect of TGF-β to completely suppress GRE activity, and markedly impair GC-inducible gene expression, is likely to contribute to glucocorticoid resistance observed in clinical settings.

The complexity of glucocorticoid molecular mechanisms is continuing to be unravelled. The simple story of transactivation of pGRE-containing genes, tethered transrepression of pro-inflammatory transcription factors, in particular NFκB and AP-1, and a couple of examples of nGRE-containing repressed genes, has long been outdated. In this study, we examined transactivational mechanisms through an examination of both GRE-dependent promoter activity and GC-inducible gene expression changes. Whilst 40pM TGF-β caused complete inhibition of GRE activity, not all GRE-containing genes are inhibited at this concentration, and those that are exhibit varying degrees of inhibition, suggestive of discordance between GRE suppression and the impairment of gene expression. This, however, is neither surprising nor unexpected. Synthetic GRE reporter constructs (and other reporter constructs) are well known to behave differently to endogenous promoters. They contain multiple tandem GRE sequences, and do not have the same complexity of regulation in terms of chromatin structure and epigenetic regulation, or the same regulation of expression by cofactors and sensitivity to post-translational modifications of GRα, as do endogenous GC inducible genes [31]. The complexity of regulation of endogenous promoters may also explain the differential effect of TGF-β on the panel of glucocorticoid-inducible genes assessed; an observation consistent between both epithelial cell lines and primary differentiated cells. The fact that TGF-β shows similar patterns of regulation of glucocorticoid-inducible gene expression in differentiated primary human bronchial epithelial cells provides strong evidence of the potential for TGF-β to regulate glucocorticoid action in situ in the airways of glucocorticoid-resistant patients.

We examined tethering transrepressional mechanisms through measuring GC regulation of inflammatory gene expression and mediator release. In the presence and absence of TGF-β, a similar degree of dexamethasone-induced repression of IL-6 mRNA, IL-8 mRNA and COX-2 mRNA in BEAS-2B cells, and COX-2 mRNA, IL-6 mRNA, IL-6 protein, and PGE_2_ production in A549 cells was observed. It has been widely accepted that glucocorticoids predominately repress the transcription of pro-inflammatory genes, such as the genes encoding COX2, IL-6 and IL-8, through a tethering transrepression of NFκB [33]. Therefore the lack of impairment by TGF-β of the glucocorticoid regulation of these genes could suggest that TGF-β has no effect on transrepressional mechanisms. In contrast, we also found that TGF-β decreased the percentage of inhibition by dexamethasone of IL-1-induced IL-8 mRNA expression in A549 cells, consistent with our previous study showing impaired IL-8 protein production in A549 cells [9] suggesting that under some conditions glucocorticoid repressional mechanisms may be impacted by TGF-β. However, the fact this impairment was not observed in the BEAS-2B cell line suggests that it may either be a cell-type-specific or stimulus-specific phenomenon, or may be due to differing direct effects of TGF-β on IL-8 expression, rather than a difference in the effect on glucocorticoid sensitivity.

Interestingly, it has been shown in A549 cells that dexamethasone does not inhibit IL-1-induced NFκB binding for up to 2 h [34] and it has further been noted that glucocorticoid effects on mRNA stability and GRE-dependent transactivation of induction of MKP-1, GILZ and IκBα, the inhibitor of NFκB can manifest as repression of gene transcription, without the involvement of transrepression mechanisms [30,31]. It is plausible therefore that the impairment of glucocorticoid regulation of IL-8 production in A549 cells by TGF-β could be a further manifestation of the impairment of transactivation process already noted. Whilst this data cannot rule out an effect of TGF-β on transrepression mechanisms, such an effect seems highly improbable given the fact that TGF-β has no effect on the glucocorticoid regulation of so many prototypical transrepressionally controlled genes.

In the A549 cell line, TGF-β decreases both GRα expression and the nuclear translocation of GRα. Therefore, the impairment of glucocorticoid activity may be, but is not necessarily, partially attributable to a deficit of GRα in the nucleus [9]. GRα protein expression is decreased by glucocorticoid treatment through the well-described process of homologous down-regulation [35]. In this study, as expected, Dex treatment caused a decrease in GRα protein expression in the BEAS-2B cell line. However, neither the basal expression of GRα, nor its susceptibility to GC-induced down-regulation were affected by TGF-β. Furthermore, the level of GRα nuclear immunoreactivity and the rate and extent of Dex-induced GRα-YFP nuclear translocation was unaffected by TGF-β, suggesting that impaired nuclear localization of GRα is not explanatory of TGF-β-induced glucocorticoid insensitivity.

The phosphorylation state of GRα is known to impact on transcriptional activity, receptor stability, association with co-activators and co-repressors, and sub-cellular localization [22]. Altered GRα phosphorylation has been associated with impaired glucocorticoid responsiveness in airway smooth muscle cells [36]. Therefore, modulation of GRα phosphorylation by TGF-β could account for the effect of TGF-β in modulating glucocorticoid activity. We found, however, that TGF-β (at a concentration that results in complete suppression of GRE activity and significant impairment of GC-inducible gene expression) has no effect on the pattern of GRα phosphorylation at Ser203, Ser211 or Ser226, the most well studied GRα phosphorylation sites. Given the relevance of GRα phosphorylation to nuclear localization, these further observations are consistent with the finding that TGF-β has no effect on GRα nuclear translocation in the BEAS-2B cell line.

We have shown in this study that inhibiting the type 1 TGF-β receptor, ALK5 in the BEAS-2B cell line is able to fully prevent the effect of TGF-β in impairing glucocorticoid transactivation, much like observations made in the A549 cell line [9]. We therefore wanted to thoroughly examine the wide variety of downstream signalling pathways activated by TGF-β, both canonical and non-canonical, to unravel the underlying mechanism through which this occurs. We targeted each of the various kinases known to be activated by TGF-β, using well-validated small molecule inhibitors at concentrations that have been widely used by us and many other groups over many years [9,15-19]. However, we were not able to even partially mimic the effect of inhibiting the ALK5 receptor through inhibiting each individual known downstream pathway. The possibility of redundancy across the signalling pathways would only be revealed by inhibiting multiple pathways simultaneously. However, combining various inhibitors together in logical and random combinations similarly had no effect on TGF-β impairment of GC action. In order to examine canonical TGF-β signalling, we chose to knockdown the expression of Smad4, the common signalling transducer of the canonical pathways. Whilst we were able to knockdown Smad4 expression by >70% and markedly impair both the activity of the Smad-dependent promoter for SM22, and the expression of PAI-1 mRNA, an endogenous Smad-regulated gene, neither GC-inducible gene expression, nor the impairment of these genes by TGF-β was affected by Smad4 knockdown. Since epigenetic regulation of gene expression has been linked to mechanisms of glucocorticoid insensitivity [37-39], we even investigated whether inhibitors of DNA methylation or histone deacetylation would prevent the GC impairment by TGF-β. However, these inhibitors similarly had no effect on TGF-β impairment of transactivation.

There are several potential explanations that remain. Firstly, TGF-β may signal through Smad2/3-dependent transcriptional activation in a Smad4-independent manner [40,41]. Therefore, our findings do not completely exclude a role for Smad-dependent signalling. The ALK5 receptor signalling pathway may also lead to downstream recruitment of known GR co-activators such as glucocorticoid receptor interacting protein 1 (GRIP-1), which has been shown to prevent glucocorticoid transactivation in airway smooth muscle cells [42], and is known to bind directly to Smad3 [43]. Similarly, TGF-β induction of GR co-repressors may mediate the transcriptional impairment observed. It is also conceivable that in our attempt to inhibit multiple signalling pathways simultaneously, we either did not inhibit the correct combination of pathways, or did not inhibit them in the correct temporal sequence for their role to be identified. Finally, and in our view the most plausible scenario is that there is a novel, non-canonical pathway being activated that could not be identified through an hypothesis-driven approach. Functional genomics or related operational approaches would therefore be required to determine the signalling cascade through which TGF-β impairs glucocorticoid action.

In this study, we have demonstrated that TGF-β potently induces insensitivity to glucocorticoid transactivation in epithelial cells, without impacting on glucocorticoid transrepression. Demonstration of the interaction of glucocorticoid and TGF-β in air-liquid interface cultures of human bronchial epithelium also raises the possibility of a mutual physiologically significant antagonistic interaction of these endogenous mediators during development, growth and repair. We have demonstrated that glucocorticoid impairment occurs downstream from the TGF-β receptor (ALK5), but is not mediated by known canonical or non-canonical pathways. Furthermore we provide data showing this impairment is not a result of epigenetic repression mechanisms, a theme that pervades recent literature concerning mechanisms of glucocorticoid resistance. This study therefore implicates novel TGF-β-inducible mechanisms as targets to modulate glucocorticoid action, and thereby restore glucocorticoid sensitivity. We consider it important to identify the downstream signalling pathways responsible due to the limitations of blocking TGF-β broadly. Since TGF-β activates such a wide variety of signalling pathways, and is involved in the homeostatic regulation of many cellular processes, it is not surprising that many unwanted side-effects develop from broadly blocking TGF-β action [44]. Of particular concern is the development of widespread inflammation and defects in autoimmunity [45,46], as well as defective haematopoiesis and other cardiovascular defects [47], demonstrated many years ago in TGF-β1 knockout mice, and more recently using small molecule ALK5 inhibitors in rats [48,49]. We believe that since TGF-β-induced glucocorticoid insensitivity appears to occur through a novel downstream signalling mechanism, it could potentially be very selectively targeted to restore glucocorticoid activity, whilst avoiding the multitude of potential side effects due to broadly inhibiting TGF-β action. Identifying this mechanism therefore has the potential to lead to new targets and novel treatments for glucocorticoid resistant disease.

## Abbreviations

ALI: Air-Liquid-Interface; COX-2: Cyclooxygenase-2; Dex: Dexamethasone; ENaCα: Epithelial sodium channel alpha subunit; GC: Glucocorticoid; GILZ: Glucocorticoid-inducible leucine zipper; GR: Glucocorticoid receptor; GRE: Glucocorticoid response element; GFP: Green fluorescent protein; HBEC: Human bronchial epithelial cells; IκBα: Inhibitor of nuclear factor kappa B; IL-6: Interleukin-6; IL-8: Interleukin-8; MAPK: Mitogen-activated protein kinase; MKP-1: MAP kinase phosphatase 1; SEAP: Secreted embryonic alkaline phosphatase; SLPI: Secretory leukocyte protease inhibitor; TGF-β: Transforming growth factor-β; YFP: Yellow fluorescent protein.

## Competing interests

All authors declare they have no competing interests.

## Authors' contributions

CK performed and analyzed all experiments using the BEAS-2B cell line and primary bronchial epithelial cells and drafted the manuscript. JM, TH, SS performed and analyzed the experiments using the A549 cell line. YX assisted with primary bronchial epithelial cell differentiation. AS assisted in interpretation of data, critically revised the manuscript and supervised the research. All authors read and approved the final manuscript.

## Supplementary Material

Additional file 1: **Live-cell, phase contrast microscopy of BEAS-2B cells transiently transfected with a GRα-YFP reporter construct.** BEAS-2B cells were treated with TGF-β (40 pM) for 24 h then imaged before and after treatment with 30 nM dexamethasone (Dex), using time-lapse live-cell fluorescence microscopy (Leica DMI6000B). Images were obtained every 30 sec for 30 min prior to Dex treatment, and for 2 h post-treatment. Click here for file
